# Psychometric Properties of the Disability Acceptance Scale in Individuals with Intellectual Disabilities

**DOI:** 10.3390/bs16101720

**Published:** 2026-09-22

**Authors:** Eun-Young Park

**Affiliations:** Department of Special Education, Jeonju University, Jeonju 55069, Republic of Korea; eunyoung@jj.ac.kr

**Keywords:** intellectual disabilities, Disability Acceptance Scale, psychometric properties

## Abstract

This study aimed to verify the psychological properties of the Disability Acceptance Scale (DAS) for individuals with intellectual disabilities using Rasch analysis. I extracted self-reported data from 216 individuals with intellectual disabilities from the Korea Panel Survey on Employment of Persons with Disabilities and performed Rasch analysis. Consequently, items 1, 3, and 7 of the original 12 items were removed as they did not meet the criteria for item fit. Subsequently, the final nine items were re-analyzed. The results revealed that all items demonstrated appropriate item fit; furthermore, item difficulty was distributed within a range capable of measuring various levels of disability acceptance. Moreover, the rating scale functioned sequentially in its response categories, and both subject (0.83) and item reliability (0.96) met the recommended standards. These results suggest that the 9-item DAS can reliably and validly evaluate the level of disability acceptance among individuals with intellectual disabilities. This study verified the metrological characteristics of the DAS for people with intellectual disabilities based on the Rasch model. The DAS can be used as an evidence-based tool for evaluating disability acceptance in education, rehabilitation, psychological counseling, and vocational rehabilitation.

## 1. Introduction

Disability entails both functional limitations and psychosocial difficulties that affect people’s emotional adaptation, self-identity, coping, and participation in rehabilitation (Marini et al., 2017). Accompanying psychological problems complicate rehabilitation and have been associated with reduced quality of life, delayed recovery, and increased incidence of secondary medical issues (Elliott & Frank, 1996). Furthermore, disability affects the lives of people with disabilities through negative emotions, such as feelings of loss, despair, and depression (Dijkers, 1997; Kreuter et al., 1998; Miller & Dishon, 2006). However, not all those with disabilities experience psychological problems. Many successfully adapt to dramatic life changes, such as destructive and damaging impairments. Studies have suggested disability acceptance as a factor explaining the emergence of these diverse adaptations (Woodrich & Patterson, 1983). The concept of disability acceptance is also referred to as adaptation to disability (Livneh, 2001).

Disability acceptance is a psychological process in which a person with a disability acknowledges and accepts their disability; furthermore, it is closely related to changes in an individual’s value system (Keany & Glueckauf, 1993). Acceptance of disability, also known as acceptance of loss, is a state in which one acknowledges that values possessed outside of the disability and the sense of loss associated with the disability no longer significantly affect their self-worth evaluation (Livneh, 2001; B. A. P. Wright, 1983). B. A. P. Wright (1983) described disability acceptance as a series of value changes, in which an individual expands their value range, decreases the value placed on physical abilities, and increases that placed on the parts that remain unchanged. Further studies elaborated on the initial concept and defined disability acceptance as shifting from comparing oneself to others to focusing on one’s other assets or abilities, expanding the value placed on impairment to other values, not extending the interpretation of impairment to all areas of life, and reducing the importance placed on one’s body or appearance (B. A. P. Wright, 1983). Disability acceptance is a process that patients must go through to become accustomed to their changed “abilities.” Thus, it is explained as a perspective of viewing oneself as “possessing other abilities” rather than “lacking abilities” (Nicholls et al., 2012).

Individuals with high levels of disability acceptance do not place excessive value on direct problems or losses caused by the disability; furthermore, they have a reduced tendency to compare themselves to non-disabled people and focus less on the negative impacts of the disability (Li & Moore, 1998). Therefore, disability acceptance—which encompasses changes in emotional responses, self-evaluation, social comparison, and personal value systems in relation to disability—significantly influences individuals’ quality of life and adaptation. This underscores disability acceptance as an important research topic in rehabilitation (Livneh & Wilson, 2003).

Linkowski (1971) conceptualized disability acceptance as an adaptive process in which an individual with a disability psychologically accepts their disability and integrates it as part of their life. Accordingly, Linkowski (1971) developed a scale comprising 50 items to measure this construct. Subsequently, to enhance the usability of the original scale, S. B. Kaiser et al. (1987) developed the Disability Acceptance Scale (DAS), which comprises nine items reflecting an individual’s values and attitudes toward disability. The DAS can concisely evaluate how individuals with disabilities perceive and accept their disabilities; it has been widely used in research to evaluate psychosocial adaptation and rehabilitation outcomes for people with various disabilities. Although alternative measurement tools such as the revised ADS-R exist (Groomes & Linkowski, 2007), they can impose a significant cognitive and linguistic burden on IWID due to their relatively large number of items and complex sentence structures. In contrast, the DAS offers clear practical advantages and differentiated additional value by concisely measuring the core areas of disability acceptance with only 12 items. This structural simplicity effectively reduces respondent fatigue and cognitive burden, making it useful for self-report surveys and large-scale panel studies involving individuals with intellectual disabilities. Importantly, disability acceptance should not be regarded as simply an attitude toward physical impairment. Rather, it involves how individuals understand their disability, evaluate themselves in relation to their disability, reorganize their personal values, and integrate disability into their broader sense of self and daily life (Groomes & Linkowski, 2007; B. A. P. Wright, 1983). This conceptualization is particularly relevant for individuals with intellectual disabilities (IWID), for whom disability may be experienced in conjunction with differences in cognitive functioning, adaptive behavior, communication, and social participation. Thus, the meaning and expression of disability acceptance may differ across disability populations, and the measurement properties of an instrument should not be assumed to be equivalent across groups.

Intellectual disability (ID) is defined as a disorder characterized by significant limitations in both intellectual functioning and adaptive behavior, with these limitations beginning during the developmental period. Adaptive behavior encompasses skills manifested in conceptual, social, and practical domains; ID diagnosis should give equal consideration to intellectual functioning and adaptive behavior. Furthermore, the developmental period is operationally defined as prior to age 22 (Schalock et al., 2021). These characteristics are relevant to the measurement of disability acceptance because they may influence how individuals understand abstract concepts, interpret self-referential statements, distinguish response categories, and report their own attitudes and experiences (Finlay & Lyons, 2001). Accordingly, the validity of a self-report measure developed or previously examined primarily in other disability populations cannot be assumed for IWID without empirical verification. From this perspective, the need to examine the DAS among IWID does not simply arise from a lack of previous research on this population. Rather, it arises from the theoretical and measurement-related possibility that the construct may be expressed and measured differently in a population with distinct cognitive, linguistic, and adaptive characteristics. Establishing the measurement properties of the DAS in IWID is therefore important for determining whether the scale items adequately represent disability acceptance and whether the response process functions as intended in this population.

Disability acceptance is a key psychosocial variable closely linked to quality of life, psychological well-being, social participation, and rehabilitation outcomes. Therefore, the DAS has been used as a representative self-report measurement tool in disability panel surveys and rehabilitation research. Since disability acceptance is an important indicator for evaluating intervention effects and identifying an individual’s level of psychosocial adaptation, its measurement tools must possess sufficient reliability and validity. Recent Consensus-based Standards for the selection of health Measurement Instruments (COSMIN) guidelines also recommend that the measurement properties of instruments be evaluated in the population and context in which they are intended to be used, assuming that validation results obtained from other groups should not be directly generalized (Mokkink et al., 2018). This principle provides a methodological basis for specifically examining the DAS in IWID. Nevertheless, studies have mainly verified the psychometric properties of the DAS using individuals with various types of disabilities or physical disabilities (Li & Moore, 1998; Rafi Yassin & Musa Al, 2021); meanwhile, research on IWID remains limited.

Previous studies have used the DAS to examine the relationship between disability acceptance and various psychosocial factors among IWID. For example, J. Kim et al. (2016) analyzed the relationships between leisure satisfaction, disability acceptance, social relationships, and life satisfaction among Korean adults with mild intellectual disabilities and reported that disability acceptance and leisure satisfaction were significant predictors of life satisfaction. In a study analyzing seven-year longitudinal data for individuals with intellectual and developmental disabilities, C. E. Lee and Oh (2026) verified trends in employment trajectories and reported that factors related to initial vocational adaptation and disability acceptance are key predictors that continuously determine the long-term employment status and quality of life of individuals with developmental disabilities. The DAS was originally developed as a measure of disability acceptance comprising three factors: self-satisfaction, de-emphasis on disability salience, and compensatory behavior (S. B. Kaiser et al., 1987). Subsequent studies have applied and validated this three-factor structure among individuals with physical disabilities (K. H. Kim, 2009; Martis et al., 2024). However, to the best of our knowledge, no study has yet systematically examined the dimensionality or internal structure of the DAS among IWID. However, these prior studies primarily used disability acceptance as a variable to analyze its relationship with other psychosocial variables and did not systematically verify that DAS functions appropriately within the ID group. In particular, sufficient verification has not been conducted regarding the psychometric characteristics of the DAS, such as item fit, internal structure and dimensionality, item difficulty, and the function of response categories. Therefore, apart from the fact that DAS has been used in existing research to study disability acceptance among IWID, further research is needed to determine whether DAS appropriately and validly measures disability acceptance in this group.

Classical Test Theory has been widely used to evaluate the reliability and validity of measurement tools. However, it has limitations; item characteristics can be influenced by the characteristics of the group being analyzed. Conversely, Item Response Theory examines item characteristics and person characteristics separately, placing them on a common latent scale. The Rasch model, which can estimate item difficulty and respondent ability on the same scale, is a widely used method for rigorously verifying the structural fit and item function of measurement tools (Bond & Fox, 2013; Tennant & Conaghan, 2007). The use of Rasch analysis is particularly appropriate for the present study for three related reasons. First, because the DAS is a self-report scale administered to IWID, it is important to determine whether each item functions appropriately across different levels of disability acceptance rather than relying only on overall reliability coefficients. Second, Rasch analysis allows for an examination of whether the response categories operate in the intended order, which is important when respondents may experience difficulty distinguishing among ordered response options. Third, Rasch analysis provides item-person maps and separation indices that allow researchers to determine whether the items adequately cover the range of disability acceptance represented in the target population. These features are particularly relevant when evaluating an existing scale in a population whose cognitive and linguistic characteristics may differ from those of the population in which the scale was originally developed.

Recently, studies have increasingly used Rasch analysis to verify the psychometric characteristics of measurement tools in disability research. For example, M. Lee et al. (2010) used the Rasch rating model to examine the construct validity of the Self-Efficacy/Social Support for Activity for Persons with Intellectual Disability (SE/SS-AID) scales among 146 IWID, demonstrating the applicability of Rasch analysis for evaluating the measurement properties of instruments in this population. Bacherini et al. (2024) validated the Beliefs About Adults with Intellectual Disabilities (BAID) scale using a Rasch model to assess physicians’ erroneous assumptions about adults with ID. More recently, Bacherini et al. (2026) used Rasch analysis to validate the Physicians’ Self-Efficacy in Caring for Adults with Intellectual Disabilities (SEC-ID) scale, which assesses physicians’ self-efficacy in providing care for adults with ID. Together, these studies illustrate the growing use of Rasch methods to examine whether measurement instruments function appropriately in disability-related contexts, including populations directly or indirectly involved with ID. Furthermore, research is being conducted to reexamine item fit, item difficulty, and response category function through Rasch analysis, even for scales previously developed based on Classical Test Theory. Rasch analysis can determine whether a scale measures a single latent characteristic and can also systematically evaluate whether each item appropriately reflects the respondent’s ability level and whether the response categories function as intended (Bond & Fox, 2013; Forget et al., 2014).

The need for Rasch analysis is particularly significant in self-report measurements targeting IWID. IWID may have difficulty understanding the meaning of items or distinguishing response categories due to their cognitive and linguistic characteristics. Furthermore, these characteristics can affect the accuracy and validity of the measurement (Maguire et al., 2023). Since it is difficult to assume that scales developed for general adults function identically for IWID, it is necessary to verify whether each item appropriately reflects the level of disability acceptance of IWID and whether the underlying factor structure of the scale is appropriate for this population. Accordingly, examining the dimensionality of the scale—as well as whether specific items are excessively difficult or difficult to understand and whether the response categories are appropriately distinguished and function as intended—is important for establishing the validity of the DAS for use with IWID (E. Y. Park, 2025). This study, therefore, aimed to examine the psychometric properties and underlying factor structure of the DAS among IWID. Exploratory factor analysis was conducted to examine the underlying dimensionality of the scale, followed by Rasch analysis to evaluate item fit, item difficulty, response category functioning, and person and item separation. Such verification provides important grounds for accurately measuring disability acceptance from the perspective of IWID and ensuring the reliability and validity of self-report data.

Therefore, this study used self-reported response data from IWID to verify the psychometric characteristics of the DAS through Rasch analysis. We aimed to verify whether the DAS is an appropriate tool for measuring disability acceptance in IWID and provide foundational data for future research on disability acceptance and the evaluation of intervention effects targeting IWID. The following hypotheses were established:

**H1.**  *The DAS will demonstrate an interpretable underlying factor structure among IWID*.

**H2.**  *The items of the DAS will demonstrate acceptable fit to the Rasch model*.

**H3.**  *The DAS items will demonstrate different levels of item difficulty, representing a meaningful range of disability acceptance*.

**H4.**  *The rating scale categories of the DAS will demonstrate appropriate functioning*.

**H5.**  *The DAS will demonstrate adequate person and item separation, indicating that the scale can distinguish individuals with different levels of disability*.

## 2. Methods

### 2.1. Data

This study used data from the Panel Survey of Employment for the Disabled (PSED), conducted by the Employment Development Institute of the Korea Employment Agency for the Disabled. The population for the second wave consisted of individuals with disabilities aged 15–64 years registered under the Disability Welfare Act. The sample frame was based on the Register of Persons with Disabilities maintained by the Ministry of Health and Welfare as of 31 December 2015. The sample was allocated and selected according to a sampling design developed by sampling experts, taking into consideration region, disability type, disability severity, age, and economic activity status. The number of eup, myeon, and dong administrative units selected for the survey was adjusted, and two-phase sampling was employed to ensure an appropriate allocation of participants across disability types and severity levels. A total of 4577 participants were recruited for the panel and participated in the first wave. Of the 4577 individuals, the sample of persons with intellectual disabilities numbered 398, from whom 226 self-report respondents were extracted. Finally, after excluding 10 individuals who did not respond to the DAS, data from 216 individuals were analyzed.

Within the framework of Rasch analysis, standard error (SE) serves as the primary metric for evaluating the reliability of item calibration. When a study ensures an adequate sample size (N), a well-distributed participant pool across item levels, and appropriate response patterns, the mean correct response rate typically ranges from 0.50 to 0.87. Because SE diminishes as sample size expands, the stability of item difficulty parameters is directly dictated by participant volume. Theoretically, B. D. Wright and Stone (1979) established that an item’s SE bounded within the Rasch model falls between (2/$\sqrt{N}$) and (3/$\sqrt{N}$}). The reliable estimation of item difficulty further requires a minimum of eight correct and eight incorrect responses per item, reflecting diverse participant characteristics. To achieve a precise difficulty estimation within ±1 logit at a 99% confidence interval (±2.6 SE), SE must not exceed approximately 0.38 logits. Translated into sample requirements, this dictates a minimum threshold of 27–61 participants for stable estimation, though a sample of 30 can suffice for exploratory or pilot investigations (B. D. Wright & Panchapakesan, 1969). Consequently, the sample size of 216 participants in this study is thoroughly adequate for conducting Rasch analysis and ensuring reliable item difficulty estimation.

The survey was conducted using Tablet PC-Assisted Personal Interviewing (TAPI). The process involved interviewers visiting the panel, asking respondents questions based on a structured questionnaire using a tablet PC, and collecting data by saving the responses to a computer. The PSED records whether the survey was answered by the participant or by a proxy respondent, allowing us to identify participants who directly provided their own responses to the DAS items. Since the DAS is a self-report measurement tool, and the construct of disability acceptance measures an individual’s own perceptions and attitudes, proxy responses carry the possibility of reflecting information different from the construct. Therefore, it was determined that using responses provided directly by the individual is appropriate for evaluating whether the DAS items adequately measure the intended construct from the perspective of IWID.

### 2.2. Measure

Disability acceptance was measured based on the DAS, developed by S. B. Kaiser et al. (1987) by shortening Linkowski’s (1971) Acceptance of Disability Scale (ADS). The DAS assesses how individuals with disabilities perceive and accept their disabilities and measures the extent to which they reconstruct their lives and values without attaching excessive significance to losses or limitations caused by their disabilities.

The PSED consists of 12 items, comprising nine items extracted from the DAS developed by S. B. Kaiser et al. (1987) and three items corresponding to the disability overcoming factors of the Self-Acceptance Test developed by Baek et al. (2001) and validated by Kang et al. (2008). The nine items of S. B. Kaiser et al.’s (1987) ADS were translated into Korean for domestic application. After undergoing a review and modification process by experts in the disability field, the items were finalized. Since then, the standardized Korean version has been continuously used in the PSED by the Korea Employment Agency for Persons with Disabilities. In this study, the standardized Korean version provided by the Panel Survey of Employment of Persons with Disabilities was used, rather than a scale separately translated or modified by the author. In addition, since the Panel Survey of Employment of Persons with Disabilities collects data through structured interviews by trained interviewers, all items were surveyed in Korean following the same procedure. Recently, Yeum (2025) verified the 12 items of the DAS included in the Panel Survey of Employment of Persons with Disabilities using a Rasch model with 3649 persons with disabilities and reported that the reliability and structural validity of the scale were generally appropriate. Although the original form of the DAS was developed a long time ago, the scale currently used in the Panel Survey of Employment of Persons with Disabilities is a Korean version that has been modified and supplemented to suit the cultural and linguistic context of persons with disabilities in Korea. It consists of items that evaluate the core concept of disability acceptance, which involves accepting a disability and positively perceiving one’s life and self. For this reason, it continues to be used as a representative tool for measuring disability acceptance in recent domestic research on persons with disabilities.

The PSED uses 12 items based on the DAS. Items include content related to an individual’s attitude toward disability, self-evaluation, and value system. It includes statements such as, “I am satisfied with my abilities and my disability does not bother me much,” “I have a disability but lack nothing in my life,” “My disability has the greatest impact on my life,” and “There are many things in life that are much more important than appearance.” Each item is rated on a 5-point Likert scale ranging from 1 (not at all) to 5 (very much so). A higher score indicates a higher level of disability acceptance. Items stated negatively are back-scored.

### 2.3. Group Differences in Disability Acceptance

Frequencies were used to show the general characteristics of people with intellectual disabilities; the extent to which they accept their disability based on these characteristics was presented using the mean and standard deviation (SD). We conducted *t*-tests and one-way ANOVAs to check for differences in disability acceptance levels based on gender, age, education level, degree of disability, and employment status. When the one-way ANOVA showed significant differences across groups, we used Bonferroni post hoc tests to see exactly which education-level groups differed from each other. Effect sizes were also calculated for statistically significant group differences. Eta squared (η^2^) was reported for omnibus ANOVA effects, and Cohen’s d was reported for significant pairwise comparisons.

### 2.4. Exploratory Factor Analysis

Exploratory factor analysis (EFA) was used to check the internal structure of the DAS. Principal axis factoring was used to extract factors (Fabrigar et al., 1999). It was theoretically assumed that the sub-factors of DAS are interrelated rather than independent concepts; therefore, oblique rotation was deemed more appropriate than orthogonal rotation. Among the oblique rotation methods, Promax was specifically selected over Direct Oblimin because it builds upon an initial orthogonal (Varimax) solution, thereby efficiently achieving a clearer, more robust, and easily interpretable simple structure while accommodating inter-factor correlations (Hendrickson & White, 1964; Fabrigar et al., 1999; Costello & Osborne, 2005). Parallel analysis was additionally conducted to determine the number of factors to retain. This method compares the eigenvalues of the actual data with those of randomly generated data, retaining only those factors for which the observed eigenvalues exceed the random eigenvalues. The number of factors was determined by considering factors with eigenvalues of 1.0 or higher and scree plots (Fabrigar et al., 1999). To check whether the factor analysis was suitable, the Kaiser–Meyer–Olkin (KMO) measure of sampling adequacy and Bartlett’s test of sphericity were conducted. If the KMO value was 0.70 or higher and Bartlett’s test was significant at the 0.05 level, we considered the data suitable for factor analysis (H. F. Kaiser & Rice, 1974).

### 2.5. Rasch Analysis

The Rasch Rating Scale Model was used to verify the psychometric characteristics of the DAS for IWID. Rasch analysis can estimate respondents’ levels of disability acceptance and item difficulty on the same logit scale and evaluate the appropriateness and measurement characteristics of items while minimizing dependence on a specific sample’s characteristics (Bond & Fox, 2013; Ridzuan et al., 2020; Tennant & Conaghan, 2007). For ease of interpretation, the Rasch person and item measures were transformed from logits to a T-score metric, with a mean of 50 and an SD of 10. Higher T-scores indicated higher levels of disability acceptance for person measures, whereas higher item measures represented greater levels of the underlying trait required to endorse the items. All Rasch analyses were performed using Winsteps version 3.60.

#### 2.5.1. Unidimensionality

Infit mean square (MNSQ), outfit MNSQ, infit standardized fit (ZSTD) indices, and outfit ZSTD were calculated to evaluate item fit. Infit MNSQ reflects the consistency of response patterns for items appropriate to the respondent’s ability level. Outfit MNSQ reflects the influence of extreme responses or outliers (Bond & Fox, 2013). In a perfect fit to the Rasch model, infit and outfit MNSQ values approach 1.0. A value greater than 1 (underfit) means the responses to the items are more irregular than the model predicts, indicating measurement errors or unexpected response patterns, while a value of less than 1 (overfit) means the item responses are too predictable, suggesting they might be redundant or offer limited information. Following Bond and Fox’s (2013) criteria, we considered a range of 0.50–1.50 as appropriate. Infit ZSTD and Outfit ZSTD express the deviation of Infit MNSQ and Outfit MNSQ from the expected value (1.0) in terms of a standard normal distribution (Z-score). A ZSTD value exceeding ±2 was considered indicative of a statistically significant misfit. For the assessment of item fit, the criteria proposed by Linacre (2006, 2010) were applied; only items with both MNSQ and ZSTD values exceeding 2.0 were determined to be unfit for the Rasch model. If unsuitable items were identified via item fit analysis, they were removed, and re-analysis was performed. Criteria for item removal were established prior to the analysis, and items judged to be underfit because both infit and outfit MNSQ values exceeded 1.50 were considered for removal. In the first Rasch analysis, only items 3 and 7 met the elimination criteria; thus, these two items were removed first. Subsequently, Rasch analysis was performed again on the remaining items. During this process, item 1 was identified as an unfit item meeting the elimination criteria and was removed. Finally, as a result of reperforming Rasch analysis, all remaining nine items met the preestablished goodness-of-fit criteria, and the item elimination procedure was concluded.

The point-measure correlation coefficient was examined to evaluate the direction and consistency of the relationship between item responses and the estimated person measures. Positive values indicate that higher levels of the underlying trait are associated with responses in the expected direction, whereas low or negative values may indicate that an item does not function consistently with the intended construct. Principal component analysis of the Rash residuals (PCAR) was performed to verify the assumption of the scale’s unidimensionality (Smith, 2002). This Rash-specific procedure evaluates residual dimensionality after the Rash model and is distinct from EFA. The recommended criterion for satisfying the unidimensionality of raw variance explained by measures is 20.0%; the criterion for the first contrast eigenvalue of residuals not explained by the model is 2.0 (Linacre, 2006).

In addition, person fit was analyzed to verify the consistency of participants’ responses. Person fit was evaluated based on infit and outfit MNSQ and ZSTD indices. The closer it is to 0, the better the fit, and the larger the absolute value, the greater the mismatch with the Rasch model. Generally, if it exceeds ±2, it suggests a statistically significant misfit. Based on the conservative standards of survey and psychometric research, participants were judged to be severely misfitted (person misfit), distorting the model’s estimates only when both their MNSQ value exceeded 2.0 and their ZSTD index exceeded 2.0 simultaneously (Linacre, 2006, 2010).

#### 2.5.2. Item Difficulty and Category Functioning

Wright maps were used to evaluate item difficulty and the scale’s measurement range (B. D. Wright & Stone, 1979). By presenting respondents’ levels of disability acceptance and item difficulty on the same scale, Wright maps allow for verifying whether items appropriately reflect respondents’ trait levels.

In addition, category functioning was analyzed to examine the appropriateness of the 5-point rating scale. It was evaluated through response frequency by category, average measure, step calibration, and infit and outfit MNSQ values (Andrich, 1996; Linacre, 2002; Linarce, 2012). In an appropriate rating scale, the average measure should increase sequentially as the response category score increases. The average measure represents the mean Rasch person measure (in logits) for respondents selecting each response category. Therefore, a sequential increase indicates that respondents choosing higher response categories have higher estimated levels of disability acceptance. In addition, step calibration between categories should demonstrate a logical order by increasing progressively across the response categories, indicating that each category represents a distinct level of the underlying latent trait. Category infit and outfit MNSQ statistics were also examined to evaluate whether each response category functioned as expected within the Rasch model. Values close to 1.0 indicate that a category functions appropriately, whereas substantially larger values suggest irregular or unpredictable use of that response category; substantially smaller values indicate overly predictable or redundant category use.

#### 2.5.3. Person and Item Separation Indices

To evaluate the scale’s reliability and discriminative power, the person separation index, the item separation index, person reliability, and item reliability were calculated. Person and item separation indices indicate how well a scale distinguishes respondents’ levels of disability acceptance and whether the hierarchy of item difficulty is stably formed, respectively. According to the criteria of Fisher (1992), separation indices of ≥1.50, 2.00, and 3.00 were interpreted as acceptable, good, and excellent, respectively. Reliability was interpreted similarly to Cronbach’s α, and a value of ≥0.80 was considered good (Andrich, 1982). Person and item strata were calculated from the respective separation indices using the formula (4G + 1)/3, where G represents the person or item separation index (Smith, 2001). Person strata indicate the number of statistically distinguishable groups of respondents with different levels of disability acceptance, whereas item strata indicate the number of statistically distinguishable groups of items with different levels of severity (Smith, 2001).

## 3. Results

### 3.1. Participants’ General Characteristics

Table 1 shows the participants’ general characteristics. Participants comprised 216 IWID [135 men (62.5%) and 81 women (37.5%)]. Average disability acceptance was slightly higher in women than in men; however, the difference was not statistically significant (t = −1.386, *p* > 0.05).

Regarding age group, the 15–29 age group was the largest with 98 participants (45.4%), followed by the 30–39, 40–49, 50–59, and 60–64 age groups with 59 (27.3%), 29 (13.4%), 26 (12.0%), and four participants (1.9%), respectively. Average disability acceptance was highest and lowest in the 15–29 and 60–64 age groups, respectively; however, the differences were not statistically significant (F = 1.186, *p* > 0.05).

Regarding educational level, high school graduates accounted for the largest number, with 115 people (53.2%). This was followed by middle and elementary school graduates, with 45 (20.8%) and 23 people (10.6%), respectively; 21 with no formal education (9.7%); and 12 being university graduates or higher (5.6%). Level of disability acceptance was relatively high in the high school and college graduate or higher group; conversely, it was lowest in the elementary school graduate group. Differences in the level of disability acceptance were statistically significant (F = 3.582, *p* < 0.01, η^2^ = 0.64). Post hoc Bonferroni comparisons indicated that participants with a high school education had significantly higher disability acceptance scores than those with an elementary school education. The effect size for this difference was moderate (Cohen’s *d* = 0.66). No other pairwise comparisons were statistically significant.

Based on degree of disability and the descriptive statistics of the DAS, participant distribution was as follows. Of the 216 participants, 39 (18.1%) were Grade 1, 70 (32.4%) were Grade 2, and 107 (49.5%) were Grade 3, which was the largest proportion. There was a slight tendency for the average disability acceptance score to be higher as the degree of disability was milder (closer to Grade 3), but the differences between the groups were not statistically significant (F = 1.320, *p* > 0.05).

Regarding employment status, 63 were employed (29.2%), and 153 were unemployed (70.8%). Average disability acceptance was higher among employed participants than unemployed participants; this difference was statistically significant (t = 2.846, *p* < 0.01, *d* = 0.43). Employed IWID had a higher level of disability acceptance than unemployed IWID.

### 3.2. EFA Results

The analysis showed that the KMO value was 0.844, and Bartlett’s test of sphericity was significant with χ^2^ (66) = 1072.844, *p* < 0.001, indicating that the data were suitable for factor analysis. Principal axis factoring and promax rotation were completed, and two factors were extracted, considering those with eigenvalues over 1.0. The analysis results showed that the eigenvalues for the first factor (4.645) and the second factor (2.243) of the actual data exceeded the 95th percentile threshold eigenvalues derived from randomly generated data (1.405 and 1.289, respectively). However, the eigenvalue for the third factor (0.917) fell below the random threshold (1.205). Consequently, this study adopted a two-factor structure as the final model. The two factors explained 49.55% of the total variance. Factor 1 included nine items, with factor loadings ranging from 0.536 to 0.795. Factor 2 included three items, with factor loadings ranging from 0.328 to 0.820 (Table 2).

### 3.3. Item Fit and Unidimensionality

Analysis of the goodness of fit of the 12 items of the DAS (Table 3) revealed that 10 items fit the Rasch model when the tolerance range for infit and outfit MNSQs was 0.50–1.50. Conversely, items 3 (infit MNSQ = 1.59, infit ZSTD = 5.6, outfit MNSQ = 1.72, outfit ZSTD = 6.5) and 7 (infit MNSQ = 1.72, infit ZSTD = 6.7, outfit MNSQ = 1.87, outfit ZSTD = 7.7) exceeded the tolerance range and were unsuitable items. Furthermore, their point-measurement correlation coefficients were low (0.13 and 0.19, respectively), indicating that they did not sufficiently reflect the latent characteristic of disability acceptance. Therefore, these two items were removed, and a re-analysis was conducted.

After items 3 and 7 were removed, Rasch analysis was reconducted on the 10 items (Table 4). The analysis results revealed that most items fit the Rasch model. However, Item 1 showed an Infit MNSQ of 1.95 (Infit ZSTD = 8.0) and an Outfit MNSQ of 2.03 (Outfit ZSTD = 8.3). As the Outfit MNSQ exceeded the acceptable range (0.50–1.50) and both the Infit and Outfit ZSTD values surpassed the acceptable threshold of ±2.0, Item 1 was deemed a misfit item. Conversely, the infit and outfit MNSQ for the remaining nine items were 0.70–1.11 and 0.71–1.05, respectively, indicating that they were all appropriate. Point-measurement correlation coefficients were 0.62–0.77—a good level that confirmed that the items appropriately measured the disability acceptance construct. Accordingly, item 1 was further removed, and the final analysis was conducted.

Items 1, 3, and 7 were identified as misfitting items because their infit and outfit MNSQ values exceeded 1.5, indicating underfit. This suggests greater response variability than expected by the Rasch model and may indicate response noise or that these items measure aspects different from the intended construct. Because all three items were reverse-scored, their potential contribution to the observed misfit was also considered, and they were removed from subsequent analyses.

The infit and outfit MNSQ of the final nine items were 0.80–1.16 and 0.80–1.20, respectively, both within the recommended range of 0.50–1.50. The infit and outfit ZSTD values ranged from −2.2 to 1.4 and from −2.2 to 1.9, respectively. Therefore, no item was classified as misfitting because no item exceeded both the MNSQ criterion (0.50–1.50) and the ZSTD criterion (±2.0). Additionally, point-measure correlation ranged from 0.66 to 0.78, indicating that all items had a good correlation with the disability acceptance construct. Therefore, the DAS, as a self-reported scale for IWID, exhibited measurement characteristics most suitable for the Rasch model when comprising nine items (Table 5).

The analysis results showed that the raw variance explained by the Rasch model was 51.4%, demonstrating excellent explanatory power significantly exceeding the recommended standard of 20.0% for unidimensionality. The unexplained variance in the first contrast was 2.4, slightly exceeding the strict unidimensionality standard of 2.0.

Analysis of the person fit of the 216 participants in this study indicated that the average infit and outfit MNSQ for all participants was 0.98, demonstrating a high degree of fit very close to 1.0—the ideal threshold of the Rasch model. It was determined that only 17 of the 216 participants (7.8%) were person misfits. This implies that random responses or cognitive errors that may occur due to the characteristics of vulnerable groups, such as IWID, were controlled at a statistically very stable level (less than 10%).

### 3.4. Item Difficulty

The item locations of the final nine items ranged from 41.20 to 57.60 logits. Items 12 (57.60 logits) and 8 (41.20 logits) had the highest and lowest item locations, respectively (Table 6).

Wright map analysis revealed that the items were distributed across various disability acceptance levels, indicating that they could measure several levels of disability acceptance (Figure 1). Additionally, respondents’ average ability score (person mean) was 56.85 logits, which was higher than the item means (50.00 logits). This suggests that the final nine items were generally easier for IWID to endorse. The item difficulty map showed a partial overlap between the distributions of respondents and items, with the range of person measures extending beyond the range of item locations, suggesting that the items did not fully cover the entire range of disability acceptance in IWID.

### 3.5. Rating Scale Functioning

The infit MNSQ (0.76–0.98) and outfit MNSQ (0.74–1.00) indices for categories 1–4 was within the 0.5–1.5 range, while category 5 showed an infit MNSQ of 2.05 and an outfit MNSQ of 1.69. Examining the suitability of the 5-point rating scale revealed that the observed averages for each category were −28.63, −14.43, 7.32, and 35.73, respectively, indicating a monotonic increase as the number of response categories increased. Thresholds between categories also increased sequentially, indicating that the response categories appropriately distinguished levels of disability acceptance (Table 7). However, category 5 demonstrated misfit, with an infit MNSQ of 2.05 and an outfit MNSQ of 1.69, exceeding the predefined acceptable criterion. This means that category 5 was not used the way the Rasch model expected, and there was more variability than expected in the responses that chose this category. In addition, category 5 was selected in only 3.0% of responses (n = 56), suggesting that the highest response category was used infrequently and less consistently than the other categories.

### 3.6. Person and Item Separation

The person separation index was 2.21 and person reliability was 0.83, indicating a good level of discriminability in distinguishing respondents according to their level of disability acceptance. The person separation index was 2.21, corresponding to a person strata of 3.28, suggesting that the DAS could statistically distinguish approximately three distinct levels of disability acceptance among respondents. The item separation index was 4.83, and item reliability was 0.96, which were high. The item separation index was 4.83, corresponding to an item strata of 6.77, indicating that the sample was sufficient to distinguish approximately seven statistically distinct levels of item severity.

## 4. Discussion

This study focused on a metrological verification of the DAS using Rasch analysis based on self-reported data from IWID. Analysis revealed that three (1, 3, and 7) of the original 12 items failed to meet the criteria for item fit. After these items were removed, the final 9-item scale demonstrated appropriate metrological characteristics regarding item fit, rating scale function, item difficulty, separability, and reliability. These findings underscore the importance of conducting psychometric evaluation when applying the DAS to a population different from that for which it was originally validated, including IWID.

Recently, scale verification using the Rasch model has been emphasized over the Classical Test Theory in psychometrics (Prieto et al., 2003). Rasch analysis can evaluate goodness of fit and measurement accuracy at the item level by analyzing the characteristics of items and respondents on the same logit scale (Massof, 2011). Since it can simultaneously verify whether an item measures a unidimensional latent trait and response categories function as intended, it is being used as a recommended analysis method for developing Patient-Reported Outcome Measures (Tennant & Küçükdeveci, 2023). Although research increasingly suggests that self-reported data from IWID can have sufficient reliability and validity, this presupposes that the items are appropriate for the respondents’ cognitive level (Kumar et al., 2025).

This study’s Rasch analysis and EFA results provided complementary evidence. Items 1, 3, and 7, identified as inappropriate in the Rasch analysis, were all back-scored items, and in the EFA, only these three items were separated into a second factor. Meanwhile, the remaining nine items were consistently loaded onto a single factor and demonstrated appropriate item functions in the Rasch analysis. Furthermore, the PCA of Rasch residuals supported overall unidimensionality, with the Rasch dimension explaining 51.4% of the total variance; the eigenvalue of the first contrast was 2.4, slightly exceeding the general standard of 2.0. However, the PCA of Rasch residuals was used as a diagnostic analysis to explore potential residual structures. It is necessary, therefore, to interpret the content commonality of the items constituting the contrast together with the fact that the eigenvalue exceeded the standard, rather than concluding multidimensionality solely because the eigenvalue exceeded the standard (Zechariah et al., 2022). In this study, all items constituting the first contrast were identified as reverse-scored items. This suggests that the second factor may reflect the wording of the reverse items or method effects that occurred during the response process, rather than being an independent dimension of disability acceptance (Suárez-Alvarez et al., 2018; van Sonderen et al., 2013). In particular, considering that IWID may have difficulty interpreting and responding to items expressed negatively or in a reverse manner (S. H. Park et al., 2019), these results can be interpreted as reflecting the cognitive burden of reverse-scored items.

These results are significant in that they demonstrate the measurement characteristics of the group of IWID when compared with the development and subsequent validation studies of the DAS. S. B. Kaiser et al. (1987) proposed that the DAS has three subfactors: self-satisfaction, de-emphasis on disability salience, and compensatory behavior quality. Subsequently, K. H. Kim (2009) applied the DAS to 238 elderly and young adults with hemiplegia and reported an overall reliability of Cronbach’s α = 0.88 and subdomain reliabilities of 0.88, 0.80, and 0.79. Meanwhile, Martis et al. (2024) also applied the three-factor structure of Kaiser et al. to individuals with acquired physical disabilities. In this study, by contrast, three back-scored items among the 12 items were separated into distinct factors, and the same items were identified as inappropriate in the Rasch analysis. However, the final nine items, excluding these items, were found to measure a single latent dimension. This result can be seen as an extension of existing research in that it directly verified the factor structure presented in previous studies within a group of IWID and presented a unidimensional measurement structure of the final nine items through EFA and Rasch analysis.

The difficulty of the final nine items was distributed over a relatively wide range, indicating that they could measure various levels of disability acceptance. The distribution of items and respondents overlapped in the Wright map, suggesting that the measurement range was generally appropriate (Boone et al., 2013b). Additionally, respondents’ average ability score (person mean) was 56.85 logits, which was higher than the item means (50.00 logits), indicating that the final nine items were generally easier for IWID to endorse. This person–item targeting pattern suggests that the DAS may be particularly sensitive to individuals with relatively lower levels of disability acceptance, as the items were somewhat easier than the overall level of disability acceptance observed in the sample. This characteristic may be clinically and practically meaningful because individuals with lower levels of disability acceptance may be those who are most likely to benefit from early identification and targeted intervention. Nevertheless, the item difficulty map showed a partial overlap between the person and item distributions, suggesting that the DAS covered a range of disability acceptance levels, although the person measures extended beyond the range of item locations. Therefore, the relatively greater sensitivity of the DAS to lower levels of disability acceptance should be considered an important characteristic of the scale when it is used with IWID.

In this study, the average measurement of the 5-point rating scale increased monotonically with increasing response categories, and thresholds between the categories were also arranged sequentially, confirming that each response category appropriately distinguishes levels of disability acceptance. However, category 5 showed suboptimal functioning because its average measure did not increase sequentially, indicating a poor fit of this category. Nevertheless, category 5 was retained because it was selected by only 3% of respondents, making its estimation relatively sensitive to the small number of observations, and because it represents the theoretically meaningful highest level of disability acceptance. Moreover, the non-sequential average measure alone was considered insufficient evidence to conclude that category 5 was substantively dysfunctional or that it should be removed. Thus, although the functioning of category 5 warrants caution, the overall pattern of category thresholds and response use provided sufficient justification for retaining the original 5-point response structure (Andrich et al., 1997) even for IWID.

Both the person (0.83) and item reliability (0.96) of the final scale exceeded the recommended standards. Person reliability refers to the ability to reliably distinguish respondents with different levels of disability acceptance; item reliability indicates that the hierarchy of item difficulty is likely to remain relatively stable even if the sample changes (Boone et al., 2013a). Person reliability means that respondents can be reliably distinguished according to their level of disability acceptance even in a new sample, and item reliability means that the hierarchy of item difficulty is likely to be maintained stably in a new sample (Bond & Fox, 2013; Linarce, 2012; Van Zile-Tamsen, 2017). When item reliability is ≥0.90 and item separation is ≥3.0, the hierarchy of items is considered very stable (Bond & Fox, 2013). In this study, both item reliability (0.96) and separation (4.83) met these criteria, confirming that the difficulty structure of the final nine items was formed very stably.

Since disability acceptance is recognized as a core psychosocial resource, accurately measuring disability acceptance is an essential process beyond merely evaluating psychological characteristics. It is crucial for predicting the mental health, quality of life, social participation, and vocational adaptation of IWID and verifying the effectiveness of related interventions. This study is significant as it presents measurement items that reflect the response characteristics of IWID based on Item Response Theory, building upon the existing DAS. The COSMIN guidelines recommend that patient-reported outcome measures used in specific groups undergo separate structural validity and metrological verification specifically for those groups (Mokkink et al., 2024). Thus, this study holds academic value as it verified the structural validity of the DAS for IWID and presented empirical evidence. The nine items identified in this study can serve as an evidence-based assessment tool applicable to various fields, such as education, rehabilitation, psychological counseling, and vocational rehabilitation, as their item suitability and reliability were verified based on self-report data from IWID.

In this study, significant differences were found in the level of disability acceptance depending on education level and employment status. In particular, post hoc tests for education level showed meaningful differences in disability acceptance between some education groups. This suggests that educational experience might be related to how people positively accept their disabilities by expanding self-understanding, communication skills, and opportunities for social participation. Also, employed groups showed higher levels of disability acceptance than unemployed groups, indicating that having a job can provide social roles and interpersonal experiences while boosting positive self-perception and sense of belonging. These results suggest that disability acceptance may be connected not only to individual psychological traits but also to environmental experiences such as education and social participation.

Furthermore, this study provides evidence for evaluating the psychometric properties of the DAS based on self-report data from IWID. Nevertheless, it also has certain limitations. First, since we used self-reported data from the Korean PSED, our results were based on IWID capable of self-reporting. Therefore, these results should be generalized with caution for individuals with severe intellectual disabilities who experience significant communication difficulties or those who have difficulty self-reporting. Future research should verify the applicability of the scale by including IWID of various functional levels and develop measurement tools that utilize easy-read formats or picture-based response systems. Second, although inappropriate items were excluded based on the Rasch analysis, we could not directly confirm why the removed items functioned inappropriately for IWID. Future research should determine how IWID understand and interpret items by conducting cognitive interviews or response process validity analyses. Third, the initial sample of 398 people with intellectual disabilities in this study was drawn from the PSED, which was based on a nationwide sample design. However, the final analysis included only 216 participants who were able to self-report and respond to the DAS. Therefore, it is difficult to say that the final sample fully represents the entire population of IWID in South Korea; thus, we need to be cautious when generalizing the results. Fourth, although this study verified metrological characteristics through Rasch analysis, it did not sufficiently verify the relationship with external criteria. Therefore, future studies should use the developed 9-item scale to verify criterion validity and predictive validity regarding various psychosocial variables, such as quality of life, mental health, self-determination, social participation, employment retention, depression, and anxiety. Fifth, category 5 did not have average scores that steadily increased with higher response options, and only about 3% of responses fell into this category; thus, it did not show ideal category functioning. However, category 5 is theoretically important as it represents the highest level of disability acceptance. We did not think there was sufficient reason to remove it based on these results alone and kept the original 5-point response scale. Future research should consider whether combining or reorganizing categories could improve the functioning of category 5.

## 5. Conclusions

This study verified the metrological characteristics of the DAS based on Rasch analysis via self-reported data from IWID. The analysis confirmed that the final 9-item scale, after excluding three items from the original 12, exhibited favorable metrological characteristics regarding item fit, rating scale function, item difficulty, and separation reliability. This provides empirical evidence demonstrating that the DAS can reliably and validly assess the level of disability acceptance in IWID. Furthermore, these results highlight the need for separate metrological verification that considers the specific characteristics of self-report scales applied to specific disability groups. The presented 9-item DAS can serve as an evidence-based assessment tool for evaluating disability acceptance and verifying intervention effects in education, rehabilitation, and counseling settings. In addition, it can be employed in future research related to the psychosocial adaptation, quality of life, social participation, and vocational rehabilitation of IWID.

## Figures and Tables

**Figure 1:**
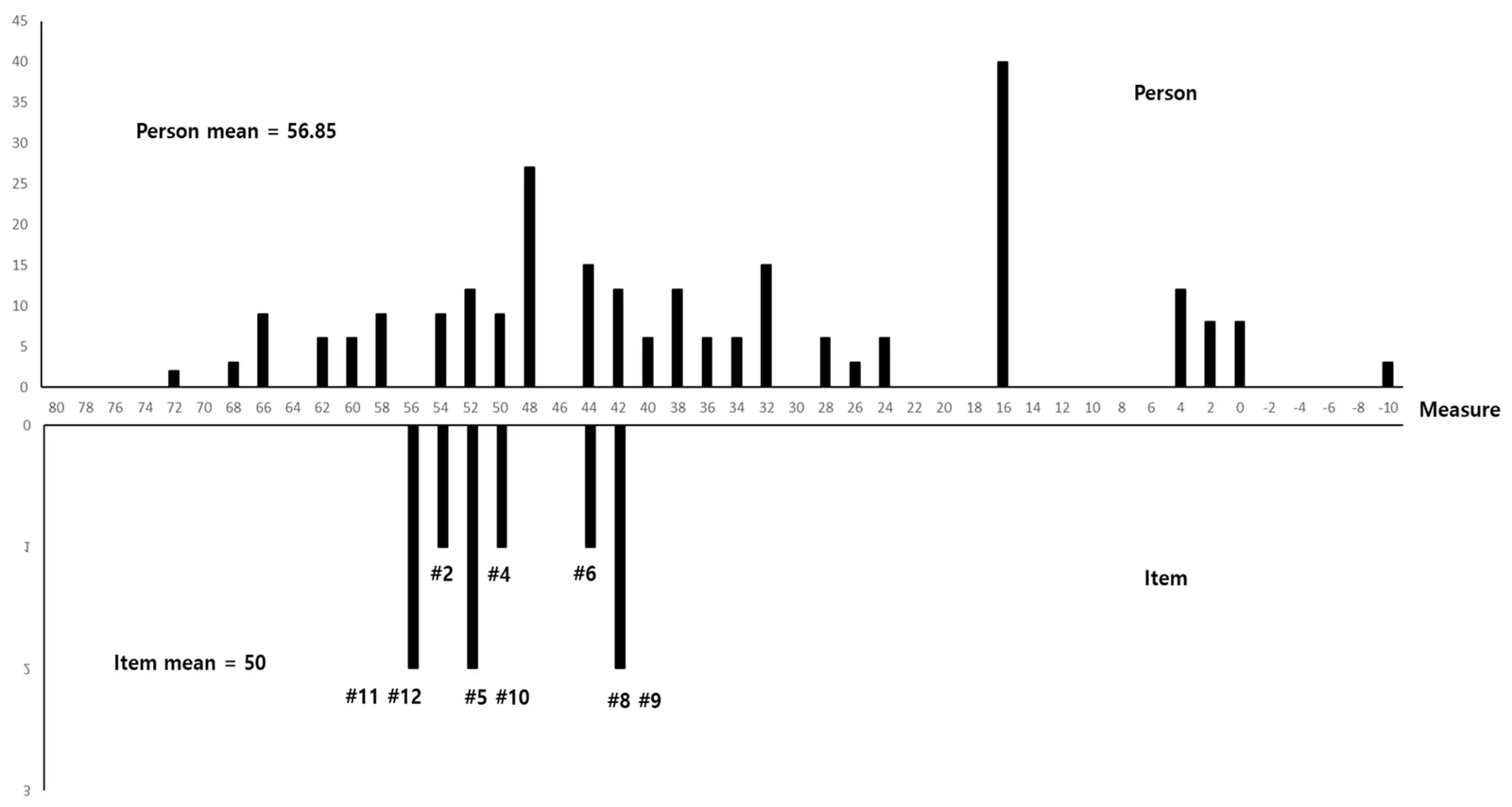
Item difficulty map.

**Table 1:** General characteristics of individuals with intellectual disabilities.

Variable	Category	n	%	DAS M	DAS SD	t/F	η^2^/d
Gender	Male	135	62.5	2.78	0.53	−1.386	
	Female	81	37.5	2.88	0.50		
Age group	15–29 years	98	45.4	2.89	0.52	1.186	
	30–39 years	59	27.3	2.77	0.51		
	40–49 years	29	13.4	2.72	0.55		
	50–59 years	26	12.0	2.80	0.78		
	60–64 years	4	1.9	2.50	0.53		
Education level	No formal education	21	9.7	2.66	0.51	3.582 **	0.64
	Elementary school	23	10.6	2.57	0.60		
	Middle school	45	20.8	2.72	0.49		
	High school	115	53.2	2.92	0.52		
	College or above	12	5.6	2.89	0.25		
Degree of disability	Grade 1	39	18.1	2.70	0.55	1.320	
	Grade 2	70	32.4	2.81	0.49		
	Grade 2	107	49.5	2.86	0.53		
Employment status	Employed	63	29.2	2.97	0.50	2.846 **	0.43
	Unemployed	153	70.8	2.75	0.52		

Note. DAS = Disability Acceptance Scale; M = mean; SD = standard deviation. ** *p* < 0.01; Intellectual disabilities are distinctively graded by IQ thresholds and adaptive capacities. Continuous, lifelong care is mandated for Grade 1 individuals, whose IQ falls below 35 alongside critical challenges in social and daily living. For Grade 2, which spans an IQ range of 35–49, individuals can acquire distinct vocational skills under ongoing supervision. By contrast, Grade 3 individuals—characterized by an IQ of 50 to 70—possess sufficient aptitude for educational and occupational rehabilitation, enabling smoother social integration.

**Table 2:** Factor structure based on a two-factor model.

Items	Factor 1	Factor 2
11. Although I have a disability, I lack nothing in my life.	0.795	0.025
5. I am disabled, but I am satisfied with my life.	0.783	−0.137
12. Although my disability causes inconvenience, I can do anything if I set my mind to it.	0.758	0.125
10. There are so many fun things to do that I forget I am disabled.	0.756	−0.012
4. I do not suffer because of my disability.	0.647	−0.053
8. Honesty is more important than the disability itself.	0.601	−0.390
9. There are many things in life that are far more important than appearance.	0.576	−0.505
6. How you live your life is more important than the disability itself.	0.545	−0.528
2. My disability has made me think about the world more broadly.	0.536	−0.199
7. My disability has the greatest impact on my life.	−0.020	0.820
3. I feel upset when there is something I cannot do because of my disability.	−0.102	0.637
1. I cannot make friends easily because I am disabled.	0.229	0.328
Eigenvalue	4.645	2.243
Percentage of Variance Explained	34.837	14.713
Explained Variance (%)	34.837	49.550

**Table 3:** Item fit statistics of the DAS (N = 216).

Item	Measure	SE	Infit MNSQ	Infit ZSTD	Outfit MNSQ	Outfit ZSTD	Pt-Measure Corr.	Decision
#1	53.56	0.84	1.36	3.6	1.36	3.6	0.44	Retain
#2	51.44	0.84	0.91	−1.0	0.90	−1.1	0.55	Retain
#3	52.42	0.84	1.59	5.6	1.72	6.5	0.13	Remove
#4	48.71	0.85	0.77	−2.8	0.76	−2.9	0.69	Retain
#5	50.39	0.85	0.62	−4.8	0.61	−4.8	0.76	Retain
#6	44.82	0.86	1.10	1.1	1.09	1.0	0.48	Retain
#7	53.45	0.84	1.72	6.7	1.87	7.7	0.19	Remove
#8	43.85	0.87	0.90	−1.1	0.93	−0.7	0.56	Retain
#9	43.91	0.87	0.97	−0.3	0.94	−0.6	0.55	Retain
#10	50.39	0.84	0.76	−2.9	0.76	−2.9	0.73	Retain
#11	53.29	0.84	0.62	−4.8	0.64	−4.5	0.77	Retain
#12	53.77	0.84	0.70	−3.8	0.70	−3.7	0.76	Retain

Note. SE = standard error; MNSQ = Infit Mean Square; ZSTD = Standardized Z-score; Pt-Measure Corr = Point-Measure Correlation.

**Table 4:** Item fit statistics of the 10 items of the DAS.

Item	Measure	SE	Infit MNSQ	Infit ZSTD	Outfit MNSQ	Outfit ZSTD	Pt-Measure Corr.	Decision
#1	56.36	1.02	1.95	8.0	2.03	8.3	0.41	Remove
#2	53.07	1.03	0.97	−0.3	1.02	0.2	0.65	Retain
#4	48.73	1.06	0.87	−1.4	0.88	−1.3	0.73	Retain
#5	50.74	1.05	0.72	−3.2	0.73	−3.1	0.77	Retain
#6	44.29	1.11	1.11	1.1	1.05	0.5	0.62	Retain
#8	41.70	1.13	0.99	0.0	0.95	−0.4	0.67	Retain
#9	42.63	1.14	1.02	0.3	0.94	−0.5	0.65	Retain
#10	51.35	1.04	0.79	−2.3	0.77	−2.6	0.76	Retain
#11	55.34	1.03	0.70	−3.5	0.71	−3.3	0.76	Retain
#12	55.80	1.02	0.87	−1.4	0.86	−1.5	0.73	Retain

Note. SE = standard error; MNSQ = Infit Mean Square; ZSTD = Standardized Z-score; Pt-Measure Corr = Point-Measure Correlation.

**Table 5:** Item fit statistics of the nine items of the DAS.

Item	Measure	SE	Infit MNSQ	Infit ZSTD	Outfit MNSQ	Outfit ZSTD	Pt-Measure Corr.
#2	54.37	1.11	1.13	1.4	1.20	1.9	0.66
#4	49.37	1.14	1.00	0.0	0.99	−0.1	0.74
#5	51.71	1.13	0.81	−2.0	0.84	−1.6	0.78
#6	44.20	1.19	1.16	1.5	1.07	0.7	0.66
#8	41.20	1.22	1.05	0.5	1.02	0.2	0.70
#9	42.21	1.22	1.14	1.4	1.06	0.6	0.67
#10	52.42	1.12	0.90	−1.0	0.87	−1.3	0.77
#11	56.91	1.10	0.80	−2.2	0.80	−2.2	0.77
#12	57.60	1.10	1.00	0.1	0.98	−0.2	0.74

Note. SE = standard error; MNSQ = Infit Mean Square; ZSTD = Standardized Z-score; Pt-Measure Corr = Point-Measure Correlation.

**Table 6:** Item difficulty estimates of the final 9-Item DAS.

Rank	Item	Measure
1	#12	57.60
2	#11	56.91
3	#2	54.37
4	#10	52.42
5	#5	51.71
6	#4	49.37
7	#6	44.20
8	#9	42.21
9	#8	41.20

**Table 7:** Appropriate of the rating scale of the final 9-Item DAS.

Category	Count	%	Observed Average	Infit MNSQ	Outfit MNSQ	Threshold
1	170	9.0	−25.13	0.98	1.00	—
2	440	23.0	−14.46	0.94	0.95	−28.63
3	776	41.0	−3.29	0.76	0.74	−14.43
4	460	24.0	9.42	0.92	0.91	7.32
5	56	3.0	12.91	2.05	1.69	35.73

Note. MNSQ = infit mean square.

## Data Availability

Data can be requested at: https://edi.kead.or.kr/Index.do (accessed on 15 September 2024).

## Abbreviations

The following abbreviations are used in this manuscript:

ADS	Acceptance of Disability Scale
BAID	Beliefs About Adults with Intellectual Disabilities
DAS	Disability Acceptance Scale
EFA	Exploratory Factor Analysis
KMO	Kaiser–Meyer–Olkin
IWID	Individuals with Intellectual Disabilities
MNSQ	Mean Square
PCA	Principal component analysis
PSED	Panel Survey of Employment for the Disabled
SD	Standard Deviation
SEC-ID	Self-Efficacy in Caring for Adults with Intellectual Disabilities
SE/SS-AID	Self-Efficacy/Social Support for Activity for Persons with Intellectual Disability
SE	Standard error
TAPI	Tablet PC-Assisted Personal Interviewing
ZSTD	Satanized Z-score
